# Risk factors for developing acute kidney injury in older people with diabetes and community-acquired pneumonia: a population-based UK cohort study

**DOI:** 10.1186/s12882-017-0566-x

**Published:** 2017-05-01

**Authors:** Anu Jain, Helen I. McDonald, Dorothea Nitsch, Laurie Tomlinson, Sara L. Thomas

**Affiliations:** Faculty of Epidemiology and Population Health, London School of Hygiene and Tropical Medicine, London, WC1E7HT UK

**Keywords:** Acute kidney injury, Diabetes, Older, Community-acquired pneumonia, UK

## Abstract

**Background:**

Acute kidney injury (AKI) is being increasingly recognised in ageing populations. There are a paucity of data about AKI risk factors among older individuals with diabetes and infections, who are at particularly high risk of AKI. The objective of this study was to evaluate the risk factors for developing acute kidney injury (AKI) amongst older patients with diabetes and community-acquired pneumonia (CAP) in England, and whether the impact of underlying kidney function varied with age.

**Methods:**

This was a population-based retrospective cohort study over 7 years (01/04/2004–31/3/2011) using electronic health records from the Clinical Practice Research Datalink linked to Hospital Episode Statistics. The study population comprised individuals with diabetes aged ≥65 years with CAP. Associations between demographic, lifestyle factors, co-morbidities and medications and development of AKI within 28 days of CAP were explored in a logistic regression model.

**Results:**

Among 3471 patients with CAP and complete covariate data, 298 patients developed subsequent AKI. In multivariable analyses, factors found to be independently associated with AKI included: male sex (adjusted odds ratio, aOR: 1.56 95% confidence interval (CI): 1.20–2.04), hypertension (aOR1.36 95% CI 1.01–1.85), being prescribed either angiotensin-converting-enzyme inhibitors or angiotensin-II-receptor-blockers (aOR: 1.59 95% CI: 1.19–2.13), or insulin (aOR: 2.27 95% CI: 1.27–4.05), presence of proteinuria (aOR 1.27 95% CI 0.98–1.63), and low estimated glomerular filtration rate (eGFR). The odds of AKI were more graded amongst older participants aged ≥80 years compared to those of younger age: for eGFR of ≤29 mL/min/1.73m^2^ (vs 60 ml/min/1.73m^2^) aOR: 5.51 95% CI 3.28–9.27 and for eGFR 30–59 mL/min/1.73m^2^ 1.96 95% CI 1.30–2.96, whilst any eGFR < 60 ml/min/1.73m^2^ was associated with approximately 3-fold increase in the odds of AKI amongst younger individuals (*p*-value for interaction = 0.007).

**Conclusions:**

The identified risk factors should help primary care and hospital providers identify high risk patients in need of urgent management including more intensive monitoring, and prevention of AKI following pneumonia.

## Background

Acute kidney injury (AKI, a term introduced for acute renal failure in 2004) is a clinical syndrome of sudden renal impairment, and is associated with adverse prognosis [1, 2]. The global incidence of AKI in hospitalised adults reported in a 2013 meta-analysis was ~22% with a mortality rate of ~24% [3]. Other adverse outcomes associated with AKI include end-stage renal disease, chronic kidney disease (CKD) and a higher risk of cardio-vascular events [4–6].

Risk factors for AKI include severe infections, diabetes, older age and CKD [7, 8]. Older individuals with diabetes are a group at particular risk of AKI owing to multiple risk factors such as age, presence of other co-morbidities including CKD, and predisposition to serious infections [9]. A common infection in this population group is community-acquired pneumonia (CAP) [10–14], and a recent review [15] reported rate ratios ranging from 1.5 to 3.1 for CAP amongst people with diabetes compared to those without diabetes. AKI triggered by infection, including both severe and non-severe CAP, carries grave prognosis with higher mortality and morbidity requiring longer hospitalisation [16–19]. The consequences of AKI in older people with diabetes who develop pneumonia could be therefore severe, with incomplete renal recovery [5, 6]. The reasons for these adverse events following AKI amongst older individuals with diabetes following pneumonia are unclear. A study did not find differences in immune marker levels amongst individuals with and without diabetes following hospitalisation for CAP [20]. However, in this study mortality was high after CAP especially in those with diabetes, and AKI was a common problem [20]. The specific risk factors for AKI in this high risk group have not been examined in previous studies [19, 21–23].

The primary objective of this study was to determine risk factors for developing AKI within 28 days of incident CAP in patients with diabetes aged ≥65 years in England. The secondary objective was to assess whether any increased risk of AKI associated with reduced estimated glomerular filtration rate (eGFR) or proteinuria varied with age. The hypothesis was that older participants with reduced eGFR may be more predisposed to AKI compared to younger individuals because of decreasing renal functional reserve [24].

## Methods

The data source for this study was the Clinical Practice Research Datalink (CPRD) containing anonymised patient records from UK general practices covering ~7% of the population and including cumulatively 79 million person-years of follow-up [25, 26]. This longitudinal and quality-assured database provides clinical, treatment, laboratory, demographic and lifestyle information for patients seen in primary care [26]. In England, 75% of CPRD general practices have consented to linkages with hospitalisation data (Hospital Episode Statistics, HES) and small-area level deprivation data (Index of Multiple Deprivation, IMD) [26]. In HES, the period between admission and discharge is known as a ‘spell’ which comprises of one or several episodes that are the periods of continuous care from a single consultant. The record for each spell is composed of a list of diagnoses occurring during each episode of the patient’s admission [27].

This study is a subset of a large retrospective cohort of older patients with diabetes, used to investigate risks associated with CKD and infections up to March 2011. Details of the overall study population and the algorithms used to diagnose CAP and other infections are published elsewhere [28, 29]. For this study, the study population comprised individuals aged ≥65 years with diabetes mellitus in England whose general practices consented to dataset linkages, and who had a first diagnosis of CAP between April 2004 and March 2011. The start date was chosen to ensure that the study followed the introduction of the Quality and Outcomes Framework in 2004 which standardised management of chronic conditions in primary care [30], and the introduction of consensus criteria for AKI (also in 2004) [31]. Individuals who had a first CAP diagnosis less than a year after they registered with the practice were excluded, as these could be historical episodes of CAP that were recorded retrospectively in the first few months after registration [32].

### Outcome and exposure data

The outcome of interest was AKI diagnosed in hospital, with the hospital episode start date within 28 days of the CAP diagnosis. This included patients who were diagnosed and treated with CAP in primary care; follow-up was not limited to hospitalisation for CAP but included any admission for AKI in the 28 days following CAP diagnosis. The primary definition of AKI was a record in the HES data coded with the specific International Classification of Diseases-10 (ICD-10) code N17 (acute renal failure). Patients with either ICD-10 codes N17 or N19 (unspecified kidney failure) were used in sensitivity analyses to capture additional patients with AKI who might have been coded with the less specific ICD-10 code.

Potential risk factors for AKI identified in previous studies were examined [19, 21, 33–37]. These included older age, gender, body mass index (BMI), deprivation (IMD), smoking status and alcohol intake; nine co-morbidities: CKD, hypertension, congestive cardiac failure, ischemic heart disease, cerebrovascular disease, dementia, chronic lung disease, malignancies, connective tissue disease and three medications: the prescription of angiotensin converting enzyme inhibitors (ACEI) and/or angiotensin II receptor blockers (ARBs) prescribed within 6 months of CAP onset, and lifetime history of anti-diabetes prescriptions (oral hypoglycaemic drugs and/or insulin) prior to infection. Socio-economic status was ascertained using 2007 Office for National Statistics’ IMD, a composite area-level marker of deprivation categorised in quintiles (1, least deprived; to 5, most deprived) [38]. Records with missing individual level IMD were substituted with values for the relevant general practice. All other risk factors were defined using primary care records in CPRD. The most recent glycated haemoglobin level that was recorded at least 28 days prior to infection onset (to describe baseline glycaemic control independent of incipient CAP) was used. BMI was estimated using the weight recorded closest to the CAP onset date and categorised into underweight (<18.5 kg/m^2^), healthy weight (18.5–24.9 kg/m^2^), overweight (25–29.9 kg/m^2^) and obese (≥30 kg/m^2^). Similarly, smoking status (non-, current or ex-smoker) and alcohol intake (non-, ex-, current up to 6 units/day and heavy >6 units/day) were ascertained using the last record prior to CAP onset and if this was unavailable then the earliest record after the infection onset were used. Diagnostic Read codes at any time prior to CAP onset date were used to identify the nine co-morbidities of interest, and prescription records at any time prior to CAP onset for medication history. Underlying renal function was ascertained from primary care records using either estimated glomerular filtration rate (eGFR, from laboratory reports) or presence of proteinuria without urinary tract infection (from laboratory reports or Read codes for proteinuria or proteinuric disease recorded by GPs), as detailed in our previous study [39]. In brief, eGFR was based on the latest serum creatinine result recorded prior to CAP onset (excluding results recorded within 28 days of CAP onset as these may reflect the effect of acute infection rather than pre-existing underlying renal function) and was estimated using the Chronic Kidney Disease Epidemiology Collaboration equation adjusted for ethnicity [40]. It was classified under three categories: ≥60, 30–59 and ≤29 mL/min/1.73m^2^ to maintain adequate numbers in each group. Proteinuria was identified by a positive urine protein test (without a concurrent urinary tract infection), or by any Read code recording proteinuric disease as previously described [39].

### Data analysis

Individuals were followed up from the CAP diagnosis date (the index date) until the earliest of: 28 days after the CAP diagnosis, AKI diagnosis, patient’s death or transfer out of the practice, the last date at which the practice contributed data, the last HES linkage date, initiation of renal replacement therapy (recorded in either CPRD or HES) or 31/03/11.

Multivariable logistic regression was used to examine the association of explanatory variables with presence or absence of an AKI-code in the 28 days after CAP, using odds ratios (OR) and 95% confidence interval (CI). Individuals with complete covariate data were included in analyses.

Age, gender and study-period were considered a priori confounders as well as potential explanatory variables in their own right. The study period split into two categories, April 2004–September 2007 and October 2007–March 2011, to assess the impact of the Acute Kidney Injury Network (AKIN) criteria introduced in 2007 [41]. The ORs for each explanatory variable were first examined in a minimally adjusted model, adjusted for the three a priori confounders. Hypothesis testing was conducted using likelihood ratio test. Ordered categorical variables such as eGFR, BMI, smoking-status, alcohol-intake and the study-period were also examined for linear trend.

**Fig. 1:**
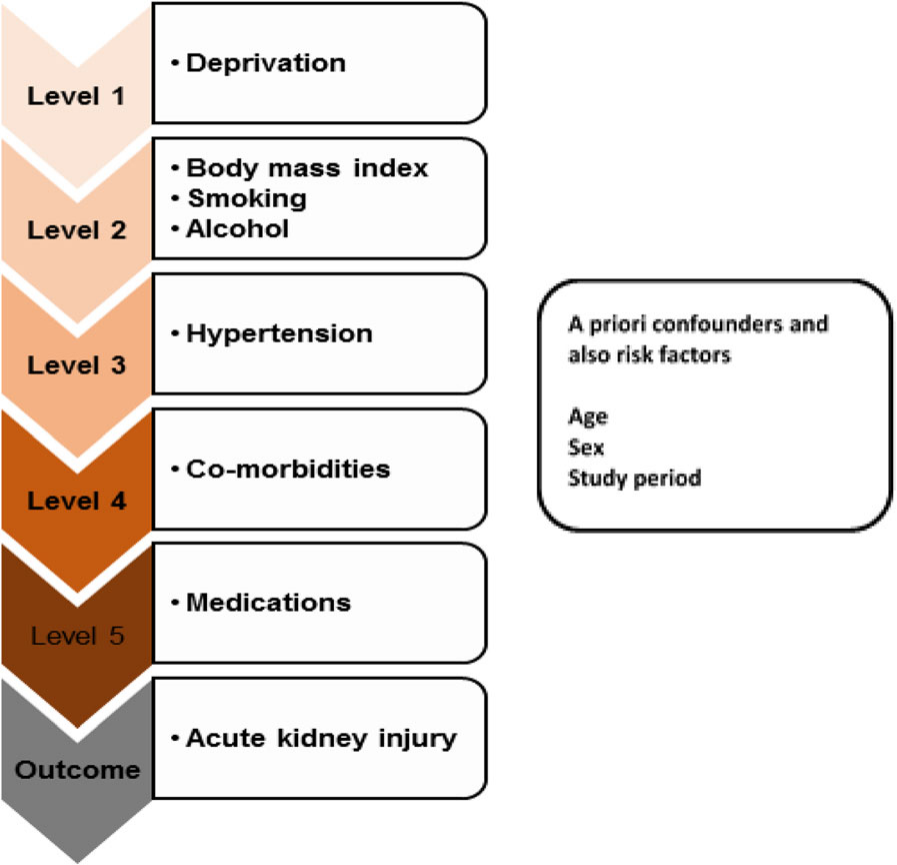
Hypothesized conceptual hierarchical framework for risk factors for acute kidney injury

A conceptual framework describing the hypothesised hierarchy of risk factors for AKI was then developed (Fig. 1) to inform the building of a multivariable model to determine risk factors for AKI [19, 21, 33, 34, 36, 37]. Deprivation (IMD) was considered a distal determinant [42], that mediates its effect first via lifestyle factors (BMI, smoking and alcohol) and then via more proximate determinants [42]: hypertension and other co-morbidities, which themselves could act partly via medications (Fig. 1). These variables were added sequentially to the minimally adjusted model in the order of their hypothesised position in the causal pathway to AKI.

Being cautious about data sparsity and collinearity, the number of variables included in the final model was restricted to ensure at least ten outcomes per parameter estimate [43]. If data sparsity was encountered the decision for inclusion in the final model was based on the magnitude of the effect estimates in the minimally adjusted model and *P*-values of <0.2. Collinearity was assessed by comparing the log standard errors of the coefficients in the multivariable models.

An interaction of a priori interest, namely between age and renal functional reserve was examined by fitting the final multivariable model first with interaction terms between age and eGFR and then between age and proteinuria. Age was re-categorised as a binary variable to examine effect modification (65–79 years and ≥80 years) and models with and without interaction terms were compared using the likelihood ratio tests.

Three sensitivity analyses were conducted: (1) an analysis including individuals who were excluded from the primary analysis because of missing data, dropping selected variables with missing data; (2) repeating analyses after categorising AKI using both ICD-10 N19 and N17 codes, to capture non-specific coding for AKI; and (3) repeating the final multivariable analyses for only those individuals who were hospitalised within 28 days (for any reason) of CAP diagnosis to minimise ascertainment bias of AKI.

## Results

The study population included a total of 4115 patients with CAP, of whom 346 (8.4%) had AKI. Data were missing for five variables (Table 1): BMI (7.6%), alcohol-intake (7.5%), HbA1C (4.7%), eGFR (1.2%) and smoking-status (0.5%); data on individual IMD were available for 91.1% of individuals, with IMD for the general practice used for the remaining 8.9%.

**Table 1:** Baseline characteristics of patients included and excluded from analyses (2004–2011)

Characteristics		Patients included in analysis		Patients excluded from analysis	
		Total *N* = 3471	With AKI = 298	Total *N* = 644	With AKI = 48
Females		1558 (44.9)	113 (37.9)	384 (59.6)	25 (52.1)
Age (years)	65–69	414 (11.9)	31 (10.4)	53 (8.2)	3 (6.3)
	70–74	587 (16.9)	39 (13.1)	66 (10.3)	4 (8.3)
	75–79	725 (20.9)	60 (20.1)	100 (15.5)	6 (12.5)
	80–84	810 (23.3)	72 (24.2)	141 (21.9)	14 (29.2)
	≥85	935 (26.9)	96 (32.2)	284 (44.1)	21 (43.8)
IMD^a^	1 (least deprived)	575 (16.6)	48 (16.1)	102 (15.8)	6 (12.5)
	2	808 (23.3)	72 (24.2)	166 (25.8)	17 (35.4)
	3	742 (21.4)	63 (21.1)	141 (21.9)	11 (22.9)
	4	756 (21.8)	67 (22.5)	124 (19.3)	7 (14.6)
	5 (most deprived)	590 (17.0)	48 (16.1)	111 (17.2)	7 (14.6)
Smoking status	Non-smoker	851 (24.5)	64 (21.5)	275 (42.7)	20 (41.7)
	Current smoker	634 (18.3)	53 (17.8)	94 (14.6)	6 (12.5)
	Ex-smoker	1986 (57.2)	181 (60.7)	253 (39.3)	20 (41.7)
	Missing	0 (0.0)	0 (0.0)	22 (3.4)	2 (4.2)
Alcohol intake u/day	Non-drinker	517 (14.9)	45 (15.1)	83 (12.9)	10 (20.8)
	Ex-drinker	530 (15.3)	53 (17.8)	38 (5.9)	4 (8.3)
	Current ≤6 units/day	2281 (65.7)	192 (64.4)	199 (30.9)	7 (14.6)
	Heavy >6 units/day	143 (4.1)	8 (2.7)	17 (2.6)	4 (8.3)
	Missing	0 (0.0)	0 (0.0)	307 (47.7)	23 (47.9)
BMI categories (kg/m^2^)	15–18.4	126 (3.6)	9 (3.0)	23 (3.6)	1 (2.1)
	18.5–24.9	1177 (33.9)	97 (32.6)	110 (17.1)	8 (16.7)
	25–29.9	1173 (33.8)	93 (31.2)	123 (19.1)	13 (27.1)
	≥30	995 (28.7)	99 (33.2)	77 (12.0)	6 (12.5)
	Missing	0 (0.0)	0 (0.0)	311 (48.3)	20 (41.7)
eGFR (mL/min/1.73m^2^)	≤29	280 (8.1)	54 (18.1)	46 (7.1)	8 (16.7)
	30–59	1487 (42.8)	165 (55.4)	262 (40.7)	19 (39.6)
	≥60	1704 (49.1)	79 (26.5)	288 (44.7)	19 (39.6)
	Missing	0 (0.0)	0 (0.0)	48 (7.5)	2 (4.2)
Proteinuria		1326 (38.2)	152 (51.0)	109 (16.9)	11 (22.9)
Hypertension		2450 (70.6)	236 (79.2)	368 (57.1)	31 (64.6)
Congestive cardiac failure		743 (21.4)	74 (24.8)	119 (18.5)	7 (14.6)
Ischaemic heart disease		1326 (38.2)	108 (36.2)	167 (25.9)	11 (22.9)
Cerebrovascular diseases		919 (26.5)	80 (26.9)	189 (29.4)	18 (37.5)
Dementia		239 (6.9)	23 (7.7)	107 (16.6)	7 (14.6)
Chronic lung disease		895 (25.8)	67 (22.5)	143 (22.2)	13 (27.1)
Cancer		638 (18.4)	46 (15.4)	112 (17.4)	6 (12.5)
Connective tissue diseases		329 (9.5)	19 (6.4)	58 (9.0)	4 (8.3)
ACEIs		1603 (46.2)	164 (55.0)	212 (32.9)	25 (52.1)
ARBs		579 (16.7)	70 (23.5)	45 (7.0)	3 (6.2)
Either ACEIs/ ARBs		2105 (60.7)	220 (73.8)	253 (39.3)	27 (56.2)
HBA1C	Good <7%	1887 (54.4)	163 (54.7)	278 (43.2)	20 (41.7)
	Borderline 7–10%	1427 (41.1)	122 (40.9)	146 (22.7)	12 (25.0)
	High >10%	157 (4.5)	13 (4.4)	25 (3.9)	4 (8.3)
	Missing	0 (0.0)	0 (0.0)	195 (30.3)	12 (25.0)
Medication-diabetes	None	859 (24.8)	64 (21.5)	270 (41.9)	18 (37.5)
	Oral	1802 (51.9)	144 (48.3)	264 (41.0)	20 (41.7)
	Insulin	142 (4.1)	22 (7.4)	43 (6.7)	4 (8.3)
	Both	668 (19.3)	68 (22.8)	67 (10.4)	6 (12.5)
Study period	April 2004- Sep.2007	1325 (38.2)	57 (19.1)	331 (51.4)	12 (25.0)
	Oct.2007-March 2011	2146 (61.8)	241 (80.9)	313 (48.6)	36 (75.0)

(%) column percentages IMD index of multiple deprivation ^a^8.9% records with missing individual index of multiple deprivation were substituted by values for general practice u units; *BMI* body mass index, *eGFR* estimated glomerular filtration rate, *ACEIs* angiotensin converting enzyme inhibitors, *ARBs* angiotensin II receptor blockers, *HBA1C* glycated-haemoglobin levels Sep. September Oct. October

The complete case analyses included 3471 patients, with 298 (8.6%) cases of AKI. Most (3047/3471 (87.8%)) of the patients with CAP were hospitalised. A comparison of patients included and excluded from analysis is shown in Table 1. Briefly, those included in analysis were more likely to be males, younger, ex-smokers, current alcohol-consumers and with raised BMI. They were also more likely to have records of proteinuria, hypertension and ischaemic heart disease and to have been prescribed ACEI or ARBs or anti-diabetic medications, but were less likely to have dementia.

### Minimally adjusted analysis

In the analysis adjusted for age, gender and study period (Table 2 column 2), there was little evidence of an association between most demographic and lifestyle factors (age, deprivation, smoking and alcohol intake) and AKI, except for increased odds of AKI amongst males and the most obese individuals (BMI ≥30 kg/m^2^). More AKI diagnoses were observed in the second half of study period. Amongst the nine co-morbidities examined as risk factors, the odds for AKI in patients with hypertension was 1.6 times higher compared to those without hypertension. There was also very strong evidence for an association with underlying renal function: there was a linear trend in increasing odds of AKI with decreasing eGFR, with those with eGFR ≤ 29 at more than five times the odds of AKI, and those with proteinuria at 1.7 times the odds of AKI. In contrast, there was some evidence for reduced odds for AKI among those with connective tissue disease and cancer. For medication, higher odds of AKI were observed amongst patients taking either ACEI/ARB, and among those prescribed insulin. Due to concerns about data-sparsity, IMD (the least strongly associated variable in this analysis) was dropped from further models.

**Table 2:** Multivariable analyses of the association of risk factors and acute kidney injury (*N* = 3471)

Characteristics		Model 1 adjusted for age, sex and period OR (95% CI)	*P* value^a^ (P_T_)	Model 4^c^ adjusted for all variables in table except IMD, medications and HBA1C OR (95% CI)	*P* value^a^ (P_T_)	Model 5 additionally adjusted for medications & HBA1C OR (95% CI)	*P* value^a^ (P_T_)
Females		0.71 (0.56–0.91)	0.007	0.63 (0.48–0.82)	0.0005	0.64 (0.49–0.83)	0.0008
Age years	65–69	0.67 (0.44–1.03)	0.11	0.92 (0.57–1.48)	0.74	0.83 (0.52–1.35)	0.55
	70–74	0.62 (0.42–0.92)		0.76 (0.50–1.17)		0.70 (0.46–1.09)	
	75–79	0.75 (0.53–1.06)		0.83 (0.57–1.20)		0.78 (0.54–1.13)	
	80–84	0.84 (0.61–1.17)		0.88 (0.63–1.24)		0.84 (0.59–1.18)	
	≥85	1		1		1	
IMD^b^	1 (least deprived)	1		Not in model		Not in model	
	2	1.09 (0.74–1.60)	0.96				
	3	1.07 (0.72–1.59)					
	4	1.16 (0.78–1.72)					
	5 (most deprived)	1.06 (0.69–1.61)					
Smoking status	Non-smoker	1		1		1	
	Current smoker	1.12 (0.76–1.67)	0.74	1.32 (0.87–2.00)	0.36	1.39 (0.92–2.11)	0.25
	Ex-smoker	1.13 (0.83–1.53)		1.22 (0.88–1.68)		1.26 (0.91–1.74)	
Alcohol intake (u/day)	Non-drinker	1		1		1	
	Ex-drinker	0.98 (0.64–1.50)	0.27	1.00 (0.65–1.55)	0.40	1.00 (0.64–1.55)	0.43
	Current ≤6 units/day	0.83 (0.59–1.18)		0.86 (0.60–1.23)		0.88 (0.61–1.26)	
	Heavy >6 units/day	0.52 (0.24–1.15)		0.56 (0.25–1.26)		0.55 (0.24–1.26)	
BMI (kg/m^2^)	15–18.4	0.80 (0.39–1.64)	0.05	0.90 (0.43–1.88)	0.50	0.95 (0.45–2.00)	0.53
	18.5–24.9	1		1		1	
	25–29.9	1.00 (0.74–1.35)		0.92 (0.67–1.25)		0.90 (0.65–1.23)	
	≥30	1.44 (1.05–1.97)		1.17 (0.85–1.62)		1.14 (0.82–1.58)	
eGFR (mL/min/1.73m^2^)	≤29	5.53 (3.75–8.15)	<0.0001	4.80 (3.18–7.23)	<0.0001	4.62 (3.06–6.99)	<0.0001
	30–59	2.74 (2.06–3.66)	(<0.0001)	2.58 (1.92–3.47)	(<0.0001)	2.48 (1.84–3.33)	(<0.0001)
	≥60	1		1		1	
Proteinuria		1.66 (1.30–2.11)	<0.0001	1.27 (0.98–1.63)	0.07	1.23 (0.95–1.59)	0.12
Hypertension		1.63 (1.21–2.19)	0.0007	1.36 (1.01–1.85)	0.04	1.27 (0.93–1.73)	0.13
Congestive cardiac failure		1.24 (0.94–1.64)	0.13	1.07 (0.79–1.45)	0.65	1.03 (0.76–1.39)	0.86
Ischaemic heart disease		0.90 (0.70–1.15)	0.4	0.79 (0.61–1.03)	0.08	0.76 (0.58–1.00)	0.04
Cerebrovascular diseases		1.00 (0.76–1.31)	0.99	0.90 (0.68–1.19)	0.44	0.90 (0.68–1.20)	0.47
Dementia		1.05 (0.66–1.65)	0.85	1.12 (0.70–1.80)	0.64	1.17 (0.73–1.88)	0.53
Chronic lung disease		0.79 (0.59–1.06)	0.11	0.79 (0.59–1.07)	0.12	0.79 (0.58–1.07)	0.12
Cancer		0.72 (0.52–1.01)	0.05	0.73 (0.52–1.03)	0.07	0.76 (0.54–1.07)	0.11
Connective tissue diseases		0.63 (0.39–1.01)	0.04	0.64 (0.39–1.05)	0.06	0.66 (0.40–1.08)	0.08
Either ACEIs/ ARBs		1.96 (1.49–2.58)	<0.0001	Not in model		1.59 (1.19–2.13)	0.002
HBA1C levels	Good <7%	1		Not in model		1	
	Borderline 7–10%	1.01 (0.79–1.30)	0.98			0.82 (0.62–1.09)	0.38
	High >7%	0.95 (0.52–1.73)				0.82 (0.43–1.54)	
Medication diabetes	None	1		Not in model		1	
	Oral	1.10 (0.81–1.50)	0.005			1.05 (0.75–1.46)	0.05
	Insulin	2.49 (1.46–4.23)				2.27 (1.27–4.05)	
	Both	1.43 (0.99–2.07)				1.11 (0.72–1.71)	
Period	April 2004- Sep. 2007	1	<0.0001	1	<0.0001	1	<0.0001
	Oct. 2007- March 2011	2.79 (2.07–3.76)	(<0.0001)	2.91 (2.14–3.94)	(<0.0001)	2.90 (2.14–3.95)	(<0.0001)

IMD index of multiple deprivation OR odds ratio CI confidence interval ^a^likelihood ratio test P_T_ *P* value for trend ^b^records with missing individual IMD were substituted by values for general practice ^c^Model 2 and 3 are presented in Table 4 in Appendix u units; *BMI* body mass index, *eGFR* estimated glomerular filtration rate, *ACEI* angiotensin converting enzyme inhibitors, *ARBs* angiotensin II receptor blockers Sep. September Oct. October

### Multivariable analysis

The multivariable models were built sequentially as described in the Methods. After adjusting for lifestyle and socio-demographic factors (Table 4 in Appendix) and then for hypertension, and then other co-morbidities (Model 4: Table 2, column 4), the increased odds associated with hypertension persisted and the linear increased odds of AKI with reducing eGFR were still observed. However, the previously observed strong evidence of higher odds of AKI with proteinuria was reduced (*P* = 0.07) and the association of high BMI with AKI seen in the previous model disappeared.

In the final multivariable analysis (Model 5: Table 2 column 6), ACEI/ARB users had 60% increased odds of AKI after adjusting for the other co-morbidities and lifestyle factors. The increased risk of AKI with insulin use remained. The previously observed association of AKI with BMI, hypertension and proteinuria was attenuated after adjusting for these additional mediating variables. There was evidence of lower odds of AKI amongst patients with ischemic heart disease.

### Effect modification by age

Table 3 shows the age-specific ORs for the association of AKI with eGFR and proteinuria as markers of CKD. There was strong evidence (P_interaction_ = 0.007) of an interaction between age and eGFR after adjusting for other risk factors for AKI, with a more pronounced effect of eGFR < 30 mL/min/1.73m^2^ on AKI amongst those aged ≥80 years (adjusted-OR 5.51 95% CI 3.28–9.27) compared to the younger group (adjusted-OR 2.90 95%CI 1.46–5.77). In the older individuals (aged ≥80 years) the graded increase in odds of AKI with decreasing eGFR remained but this graded association was not evident in the younger age-group for whom any eGFR < 60 ml/min/1.73m^2^ was associated with an approximately three fold increase in AKI risk (Table 3).

**Table 3:** Association of pre-existing eGFR and proteinuria with acute kidney injury stratified by age (*N* = 3471)

	**eGFR (mL/min/1.73m** ^**2**^ **)**	**Total**	**With AKI** **N (%)**	**Stratum-specific adjusted** ^a^ **OR for acute kidney injury (95%CI)**	***P*** **-value for interaction** ^b^
Age 65–79	≤29	102	13 (12.8)	2.90 (1.46–5.77)	0.007
	30–59	586	74 (12.6)	3.29 (2.20–4.94)	
	≥60	1038	43 (4.1)	1	
	Total	1726	130 (7.5)		
Age 80 or above	≤29	178	41 (23)	5.51 (3.28–9.27)	
	30–59	901	91 (10.1)	1.96 (1.30–2.96)	
	≥60	666	36 (5.4)	1	
	Total	1745	168 (9.6)		
	**Proteinuria**	**Total**	**With AKI** **N (%)**	**Stratum-specific adjusted** ^a^ **OR for acute kidney injury (95%CI)**	***P*** **-value for interaction** ^b^
Age 65–79	No	1057	57 (5.4)	1	0.34
	Yes	669	73 (10.9)	1.42 (0.96–2.08)	
	Total	1726	130 (7.5)		
Age 80 or above	No	1088	89 (8.2)	1	
	Yes	657	79 (12)	1.11 (0.79–1.55)	
	Total	1745	168 (9.6)		

AKI acute kidney injury (%) row percentage eGFR estimated glomerular filtration rate ^a^Model 5 in Table 2

OR odds ratio CI confidence interval ^b^likelihood ratio test

In contrast, there was little evidence (P_interaction_ = 0.34) that the effect of proteinuria on AKI varied by age.

### Sensitivity analyses (Tables 5, 6 and 7 in Appendix)

After excluding the four variables with missing data that had less strong evidence of an association with AKI (smoking status, alcohol intake, BMI and HbA1C) there were 4067 individuals included in the analysis. The results from this model were similar to those of the main model (Table 5 in Appendix).

After widening the definition of AKI to include the non-specific N19 code, the number of patients who developed AKI increased from 298 (coded only as ICD-10 N17) to 373 (coded either ICD-10 N17 or N19). The lower odds associated with connective tissue diseases was no longer apparent in this model, but no other appreciable changes were evident (Table 6 in Appendix).

Amongst those hospitalised the overall risk of developing AKI was 9.8% (*N* = 298). Results after restricting analyses to those who were hospitalised were very similar to those in the entire study population (Table 7 in Appendix).

## Discussion

Amongst older individuals with diabetes and CAP the overall risk for AKI within 28 days of incident CAP diagnosis was 8.4% for the entire cohort and 9.8% amongst those who were hospitalised. The risk factors independently associated with AKI were male sex, reduced eGFR, absence of ischemic heart disease, use of ACEIs/ARBs and insulin. The association of hypertension and proteinuria with AKI attenuated after adjusting for ACEIs/ARBs use. The association of reducing eGFR with AKI was found to be more prominent amongst individuals aged ≥80 years, whilst any eGFR < 60 ml/min/1.73m^2^ was associated with AKI risk amongst younger study participants.

The risk of 9.8% found in this cohort is lower than that reported by a Scottish study [23] that found AKI incidence of 18% amongst CAP patients at time of hospital admission. Another study [19] reported AKI incidence of 34% any time during hospitalisation in CAP patients aged ≥18 years. In both these studies the diagnosis of CAP was confirmed in the hospital and were not confined to individuals with diabetes [19, 23]. In the present study CAP diagnosis in general practice (which is mainly a clinical diagnosis) could have led to a higher study denominator and perhaps an underestimation of the risk of AKI.

A temporal change in increased AKI diagnosis during the period of 2007–2011, as observed in previous studies using CPRD and HES [44, 45] was also evident in this study, reflecting better ascertainment of outcome in the latter period. In the earlier period of the study, coded AKI events are likely to have been for the more serious forms of AKI [46]. This is not necessarily a draw-back as the analyses identifies those at highest risk of more severe AKI. The validation of recording of AKI (during 2005 and 2010) in UK hospital data has been previously described [44] with a high positive predictive value (95%) for AKI codes in these data.

The association of two markers of CKD: reducing eGFR and presence of proteinuria were identified as independent risk factors for AKI as previously reported for the general population [47, 48]. We found that age modified the strength of association of reduced eGFR with AKI risk: amongst study participants <80 years of age any reduced eGFR was associated with AKI risk, whilst amongst older study participants those with eGFR < 30 ml/min/1.73m^2^ were especially at risk of AKI. Once eGFR was added to the model, age no longer had a statistically independent association, except through the observed effect modification. There was no evidence of interaction of age with proteinuria as has been described previously for the general population [48]. Other risk factors identified in this study with AKI included male gender, BMI ≥30 kg/m^2^, hypertension and use of ACEIs & ARBs, as previously reported for the general population [8, 37, 49], for individuals with diabetes [33] and amongst patients with infections and septic shock [36, 45]. Amongst patients on insulin without oral antiglycaemic medications, it could be hypothesized that patients taking insulin alone were more likely to have Type-I diabetes with a longer disease duration affecting renal-vasculature. However, reliable data on disease duration or type were not available in this dataset and so these aspects of diabetes could not be explored in this study.

This was a population based large cohort study using quality-assured data from the UK general practices spanning a 7-year study period and describes for the first time the factors associated with AKI in this patient population. The use of conceptual framework allowed explanation of possible mediating pathways between risk factors and AKI and independent assessment of the association of both eGFR and history of proteinuria with AKI. The results are potentially generalizable to older patients with diabetes and CAP from other similar-income countries to England with similar demographic profile and access to healthcare.

This study has the limitations from using routinely collected data. Due to the nature of dataset the association of severity of CAP with AKI could not be assessed.

Another potential limitation was missing data; complete data were available for 84.3% participants and a sensitivity analysis conducted to minimise the effect of missing data using data from 98.8% of these participants showed no noticeable differences from the primary analyses. Other potential risk factors such as ethnicity, hyperlipidaemia, duration of diabetes and antibiotic use were not measured so the effect-estimates may have residual confounding. Competing risk of death may have played a role as approximately 30% of study participants died within 28 days after the CAP diagnosis [50]. Because an eGFR of <30 ml/min/1.73m^2^ is known to be associated with death after admission, the associations seen in this analysis are likely to have been underestimated for the lower eGFR risk group, and could be potentially even higher than we have shown [50]. A competing risk approach would have been particularly appropriate if we had wanted to develop a predictive risk score. However, at this stage we wished simply to understand causality (potential risk factors in this high risk group) [51]. We did not have data on the presentation of CAP – the hospital coding variables depend on coding in discharge letters and the clinical presentation is not captured by the diagnostic ICD10 codes in the HES database. We did not use the latest available serum creatinine prior to CAP diagnosis, as we wanted to capture the underlying renal function irrespective of the context of the acute infection, and because test results may not have been available for every patient except for those who were most ill. Most people with diabetes require careful adaptation of diabetes drugs in the context of altered glucose control during infection. These data are observational and the association seen with ACEIs/ARBs may be due to underlying confounding by indication. Those who are prescribed these drugs have typically higher blood pressure, or are more proteinuric, or have heart failure [52]. The standard ACEIs/ARBs prescribed in primary care have a long pharmacological half life [53]; stopping these in the context of infection means that these drugs may stay up to 7 days in the body. Therefore, whilst it is true that people who are on these drugs are at higher risk, it is not clear whether sick-day rules that advise stopping the drugs would indeed prevent AKI in the week following infection. Evaluation of this via a clinical trial would help to elucidate the potential benefits of stopping ACEIs/ARBs in this setting.

## Conclusions

In summary, amongst older people with diabetes who are diagnosed with CAP, risk of AKI is largely dependent on pre-existing renal function with age modifying this relationship, and prescription of insulin and/or ACE-I/ARB, as well as male sex. The identified risk factors should help primary care and hospital providers identify those patients with CAP who should be monitored more closely (for example, with timely administration of antibiotics and appropriate fluid management) to prevent AKI following pneumonia.

## Appendix

**Table 4:** Multivariable analyses of the association between risk factors and acute kidney injury: three Models (*N* = 3471)

Characteristics		Model 1 Adjusted for age, sex and period OR (95% CI)	*P* value^a^ (P_T_)	Model 2 additionally, adjusted for BMI, smoking, alcohol-intake OR (95% CI)	*P* value^a^ (P_T_)	Model 3 additionally adjusted for hypertension OR (95% CI)	*P* value^a^ (P_T_)
	Females	0.71 (0.56–0.91)	0.007	0.69 (0.53–0.89)	0.004	0.66 (0.51–0.86)	0.002
Age categories years	65–69	0.67 (0.44–1.03)	0.11	0.59 (0.37–0.92)	0.02	0.59 (0.37–0.92)	0.03
	70–74	0.62 (0.42–0.92)		0.55 (0.36–0.83)		0.55 (0.36–0.83)	
	75–79	0.75 (0.53–1.06)		0.68 (0.48–0.97)		0.66 (0.46–0.94)	
	80–84	0.84 (0.61–1.17)		0.80 (0.58–1.12)		0.78 (0.56–1.09)	
	≥85	1		1		1	
Estimated IMD^b^	1 (least deprived)	1		Not in model		Not in model	
	2	1.09 (0.74–1.60)	0.96				
	3	1.07 (0.72–1.59)					
	4	1.16 (0.78–1.72)					
	5 (most deprived)	1.06 (0.69–1.61)					
Smoking status	Non-smoker	1		1		1	
	Current smoker	1.12 (0.76–1.67)	0.74	1.17 (0.78–1.74)	0.68	1.18 (0.79–1.77)	0.65
	Ex-smoker	1.13 (0.83–1.53)		1.14 (0.83–1.55)		1.14 (0.83–1.56)	
Alcohol intake (units/day)	Non-drinker	1		1		1	
	Ex-drinker	0.98 (0.64–1.50)	0.27	0.97 (0.63–1.49)	0.29	0.96 (0.63–1.48)	0.24
	Current ≤6 units/day	0.83 (0.59–1.18)		0.83 (0.58–1.18)		0.82 (0.58–1.17)	
	Heavy >6 units/day	0.52 (0.24–1.15)		0.53 (0.24–1.17)		0.50 (0.23–1.11)	
BMI (kg/m^2^)	15–18.4	0.80 (0.39–1.64)	0.05	0.80 (0.39–1.63)	0.06	0.80 (0.39–1.64)	0.09
	18.5–24.9	1		1		1	
	25–29.9	1.00 (0.74–1.35)		1.00 (0.74–1.36)		0.99 (0.73–1.34)	
	≥30	1.44 (1.05–1.97)		1.43 (1.04–1.95)		1.39 (1.01–1.90)	
eGFR (mL/min/1.73m^2^)	≤29	5.53 (3.75–8.15)	<0.0001	Not in model		Not in model	
	30–59	2.74 (2.06–3.66)	(<0.0001)				
	≥60	1					
Proteinuria		1.66 (1.30–2.11)	<0.0001	Not in model		Not in model	
Hypertension		1.63 (1.21–2.19)	0.0007	Not in model		1.62 (1.21–2.18)	0.0009
Congestive cardiac failure		1.24 (0.94–1.64)	0.13	Not in model		Not in model	
Ischaemic heart disease		0.90 (0.70–1.15)	0.4	Not in model		Not in model	
Cerebrovascular diseases		1.00 (0.76–1.31)	0.99	Not in model		Not in model	
Dementia		1.05 (0.66–1.65)	0.85	Not in model		Not in model	
Chronic lung disease		0.79 (0.59–1.06)	0.11	Not in model		Not in model	
Cancer		0.72 (0.52–1.01)	0.05	Not in model		Not in model	
Connective tissue disease		0.63 (0.39–1.01)	0.04	Not in model		Not in model	
Either ACEIs/ ARBs		1.96 (1.49–2.58)	<0.0001	Not in model		Not in model	
HbA1C	Good <7%	1		Not in model		Not in model	
	Borderline 7–10%	1.01 (0.79–1.30)	0.98				
	High >7%	0.95 (0.52–1.73)					
Medication diabetes	None	1		Not in model		Not in model	
	Oral	1.10 (0.81–1.50)	0.005				
	Insulin	2.49 (1.46–4.23)					
	Both	1.43 (0.99–2.07)					
Study period	April2004-September 2007	1	<0.0001	1	<0.0001	1	<0.0001
	October2007-March 2011	2.79 (2.07–3.76)	(<0.0001)	2.78 (2.06–3.75)	(<0.0001)	2.72 (2.01–3.66)	(<0.0001)

OR odds ratio CI confidence interval ^a^likelihood ratio test P_T_ *P* value for trend IMD index of multiple deprivation ^b^records with missing individual IMD were substituted by values for general practice u/d units/day BMI body mass index eGFR estimated glomerular filtration rate ACEI angiotensin converting enzyme inhibitors ARBs angiotensin II receptor blockers

**Table 5:** Sensitivity analysis 1: Association of risk factors and acute kidney injury: multivariable analysis excluding smoking status, alcohol intake, body mass index and glycated-haemoglobin levels (*N* = 4067, outcome = 344)

Characteristics		Model from primary analysis^a^ Odds ratio (95% confidence interval)	*P* value^c^ (P_T_)	Sensitivity analysis Odds ratio^b^ (95% confidence interval)	*P* value^c^ (P_T_)
Females		0.64 (0.49–0.83)	0.0008	0.64 (0.51–0.82)	0.0003
Age categories (years)	65–69	0.83 (0.52–1.35)	0.55	0.85 (0.55–1.31)	0.64
	70–74	0.70 (0.46–1.09)		0.75 (0.51–1.12)	
	75–79	0.78 (0.54–1.13)		0.82 (0.58–1.15)	
	80–84	0.84 (0.59–1.18)		0.93 (0.68–1.26)	
	≥85	1		1	
Smoking status	Non-smoker	1		Not in model	
	Current smoker	1.39 (0.92–2.11)	0.25		
	Ex-smoker	1.26 (0.91–1.74)			
Alcohol intake (units/day)	Non-drinker	1		Not in model	
	Ex-drinker	1.00 (0.64–1.55)	0.43		
	Current ≤6 units/day	0.88 (0.61–1.26)			
	Heavy >6 units/day	0.55 (0.24–1.26)			
BMI (kg/m^2^)	15–18.4	0.95 (0.45–2.00)	0.53	Not in model	
	18.5–24.9	1			
	25–29.9	0.90 (0.65–1.23)			
	≥30	1.14 (0.82–1.58)			
eGFR (mL/min/1.73m^2^):	≤29	4.62 (3.06–6.99)	<0.0001	4.43 (3.03–6.46)	<0.0001
	30–59	2.48 (1.84–3.33)	(<0.0001)	2.21 (1.68–2.90)	(<0.0001)
	≥60	1		1	
Proteinuria		1.23 (0.95–1.59)	0.12	1.22 (0.96–1.56)	0.10
Hypertension		1.27 (0.93–1.73)	0.13	1.17 (0.89–1.55)	0.26
Congestive cardiac failure		1.03 (0.76–1.39)	0.86	0.97 (0.73–1.29)	0.83
Ischaemic heart disease		0.76 (0.58–1.00)	0.04	0.77 (0.60–0.98)	0.03
Cerebrovascular diseases		0.90 (0.68–1.20)	0.47	0.96 (0.74–1.24)	0.74
Dementia		1.17 (0.73–1.88)	0.53	1.10 (0.73–1.67)	0.65
Chronic lung disease		0.79 (0.58–1.07)	0.12	0.90 (0.68–1.18)	0.43
Cancer		0.76 (0.54–1.07)	0.11	0.73 (0.53–1.01)	0.05
Connective tissue diseases		0.66 (0.40–1.08)	0.08	0.68 (0.43–1.07)	0.08
Either ACEI/ARBs		1.59 (1.19–2.13)	0.002	1.66 (1.27–2.17)	0.0001
HBA1C levels	Good <7%	1		Not in model	
	Borderline 7–10%	0.82 (0.62–1.09)	0.38		
	High >7%	0.82 (0.43–1.54)			
Medication diabetes	None	1		1	
	Oral	1.05 (0.75–1.46)	0.05	1.00 (0.74–1.33)	0.08
	Insulin	2.27 (1.27–4.05)		1.89 (1.14–3.11)	
	Both	1.11 (0.72–1.71)		1.03 (0.72–1.47)	
Study period	April 2004–September 2007	1	<0.0001	1	<0.0001
	October 2007–March 2011	2.90 (2.14–3.95)	(<0.0001)	2.94 (2.22–3.89)	(<0.0001)

^a^adjusted for age, sex, study period, smoking status, alcohol intake, BMI, comorbidities, medications and glycated-haemoglobin levels ^b^adjusted for all variables in the table except smoking, alcohol intake, BMI and glycated haemoglobin levels^c^likelihood ratio test P_T_ *P* value for trend BMI body mass index eGFR estimated glomerular filtration rate ACEI angiotensin converting enzyme inhibitors ARBs angiotensin II receptor blockers

**Table 6:** Sensitivity analysis 2: Risk factors associated with acute kidney injury multivariable model with ICD10-N17 or N19 code for outcome (*N* = 3471 outcome = 373)

Characteristics		Model from primary analysis^a^ OR (95% CI)	*P* value^b^ (P_T_)	Sensitivity analysis Odds Ratio^a^ (95% confidence interval)	*P* value^b^ (P_T_)
Females		0.64 (0.49–0.83)	0.0008	0.63 (0.49–0.80)	0.0002
Age categories years	65–69	0.83 (0.52–1.35)	0.55	1.00 (0.65–1.54)	0.52
	70–74	0.70 (0.46–1.09)		0.75 (0.50–1.11)	
	75–79	0.78 (0.54–1.13)		0.82 (0.58–1.15)	
	80–84	0.84 (0.59–1.18)		0.90 (0.66–1.23)	
	≥85	1		1	
Smoking status	Non-smoker	1	0.25	1	0.32
	Current smoker	1.39 (0.92–2.11)		1.32 (0.91–1.92)	
	Ex-smoker	1.26 (0.91–1.74)		1.19 (0.89–1.59)	
Alcohol intake (units/day)	Non-drinker	1	0.43	1	0.18
	Ex-drinker	1.00 (0.64–1.55)		1.17 (0.78–1.76)	
	Current ≤6 units/day	0.88 (0.61–1.26)		0.99 (0.71–1.39)	
	Heavy >6 units/day	0.55 (0.24–1.26)		0.54 (0.25–1.16)	
BMI (kg/m^2^)	15–18.4	0.95 (0.45–2.00)	0.53	0.80 (0.40–1.61)	0.28
	18.5–24.9	1		1	
	25–29.9	0.90 (0.65–1.23)		0.84 (0.63–1.11)	
	≥30	1.14 (0.82–1.58)		1.10 (0.81–1.47)	
eGFR (mL/min/1.73m^2^)	≤29	4.62 (3.06–6.99)	<0.0001	4.86 (3.34–7.09)	<0.0001
	30–59	2.48 (1.84–3.33)	(<0.0001)	2.47 (1.89–3.23)	(<0.0001)
	≥60	1		1	
Proteinuria		1.23 (0.95–1.59)	0.12	1.22 (0.96–1.54)	0.10
Hypertension		1.27 (0.93–1.73)	0.13	1.19 (0.90–1.57)	0.21
Congestive cardiac failure		1.03 (0.76–1.39)	0.86	1.05 (0.80–1.39)	0.71
Ischaemic heart disease		0.76 (0.58–1.00)	0.04	0.77 (0.60–0.98)	0.03
Cerebrovascular disease		0.90 (0.68–1.20)	0.47	0.81 (0.62–1.05)	0.10
Dementia		1.17 (0.73–1.88)	0.53	1.17 (0.76–1.82)	0.48
Chronic lung disease		0.79 (0.58–1.07)	0.12	0.75 (0.57–0.98)	0.03
Cancer		0.76 (0.54–1.07)	0.11	0.89 (0.66–1.19)	0.42
Connective tissue diseases		0.66 (0.40–1.08)	0.08	0.73 (0.47–1.12)	0.14
Either ACEI/ ARBs		1.59 (1.19–2.13)	0.002	1.48 (1.14–1.92)	0.003
Glycated haemoglobin levels	Good <7%	1		1	
	Borderline 7–10%	0.82 (0.62–1.09)	0.38	0.85 (0.66–1.10)	0.42
	High >7%	0.82 (0.43–1.54)		1.03 (0.60–1.77)	
Medication-diabetes	None	1		1	
	Oral	1.05 (0.75–1.46)	0.05	1.02 (0.75–1.38)	0.02
	Insulin	2.27 (1.27–4.05)		2.27 (1.34–3.84)	
	Both	1.11 (0.72–1.71)		1.10 (0.74–1.62)	
Study period	April 2004–September 2007	1	<0.0001	1	<0.0001
	October 2007–March 2011	2.90 (2.14–3.95)	(<0.0001)	2.38 (1.83–3.10)	(<0.0001)

^a^adjusted for all variables in table BMI body mass index eGFR estimated glomerular filtration rate ACEI angiotensin converting enzyme inhibitors ARBs angiotensin II receptor blockers^b^ likelihood ratio test P_T_ *P* value for trend

**Table 7:** Sensitivity analysis 3: Risk factors associated with acute kidney injury multivariable model restricted to hospitalised patients (*N* = 3047 outcome = 298)

Characteristics		Primary analysis OR (95% CI)^b^	*P* value^a^ (P_T_)	Sensitivity analysis OR (95% CI)^b^	*P* value^a^ (PT)
Gender	Female	0.64 (0.49–0.83)	0.0008	0.64 (0.49–0.84)	0.001
Age categories years	65–69	0.83 (0.52–1.35)	0.55	0.84 (0.52–1.37)	0.49
	70–74	0.70 (0.46–1.09)		0.70 (0.45–1.09)	
	75–79	0.78 (0.54–1.13)		0.75 (0.52–1.10)	
	80–84	0.84 (0.59–1.18)		0.82 (0.58–1.16)	
	≥85	1		1	
Smoking status	Non-smoker	1	0.25	1	0.32
	Current smoker	1.39 (0.92–2.11)		1.34 (0.88–2.04)	
	Ex-smoker	1.26 (0.91–1.74)		1.25 (0.90–1.73)	
Alcohol intake (units/day)	Non-drinker	1	0.43	1	0.59
	Ex-drinker	1.00 (0.64–1.55)		1.00 (0.64–1.56)	
	Current ≤6 units/day	0.88 (0.61–1.26)		0.88 (0.61–1.28)	
	Heavy >6 units/day	0.55 (0.24–1.26)		0.61 (0.27–1.40)	
BMI (kg/m^2^)	15–18.4	0.95 (0.45–2.00)	0.53	1.00 (0.47–2.13)	0.56
	18.5–24.9	1		1	
	25–29.9	0.90 (0.65–1.23)		0.89 (0.65–1.22)	
	≥30	1.14 (0.82–1.58)		1.12 (0.80–1.56)	
eGFR (mL/min/1.73m^2^)	≤29	4.62 (3.06–6.99)	<0.0001	4.54 (2.98–6.90)	<0.0001
	30–59	2.48 (1.84–3.33)	(<0.0001)	2.40 (1.78–3.25)	(<0.0001)
	≥60	1		1	
Proteinuria		1.23 (0.95–1.59)	0.12	1.19 (0.92–1.55)	0.19
Hypertension		1.27 (0.93–1.73)	0.13	1.30 (0.95–1.78)	0.09
Congestive cardiac failure		1.03 (0.76–1.39)	0.86	1.06 (0.78–1.43)	0.73
Ischaemic heart disease		0.76 (0.58–1.00)	0.04	0.75 (0.57–0.98)	0.03
Cerebrovascular disease		0.90 (0.68–1.20)	0.47	0.89 (0.67–1.19)	0.44
Dementia		1.17 (0.73–1.88)	0.53	1.26 (0.78–2.04)	0.36
Chronic lung disease		0.79 (0.58–1.07)	0.12	0.76 (0.56–1.03)	0.08
Cancer		0.76 (0.54–1.07)	0.11	0.77 (0.55–1.09)	0.13
Connective tissue diseases		0.66 (0.40–1.08)	0.08	0.64 (0.39–1.05)	0.07
Either ACEI/ ARBs		1.59 (1.19–2.13)	0.002	1.57 (1.17–2.11)	0.002
Glycated haemoglobin levels	Good <7%	1		1	
	Borderline 7–10%	0.82 (0.62–1.09)	0.38	0.83 (0.62–1.10)	0.40
	High >7%	0.82 (0.43–1.54)		0.82 (0.43–1.56)	
Medication-diabetes	None	1	0.05	1	0.03
	Oral	1.05 (0.75–1.46)		1.05 (0.75–1.48)	
	Insulin	2.27 (1.27–4.05)		2.43 (1.34–4.40)	
	Both	1.11 (0.72–1.71)		1.11 (0.72–1.71)	
Study period	April 2004–September 2007	1	<0.0001	1	<0.0001
			(<0.0001)		
	October 2007–March 2011	2.90 (2.14–3.95)	(<0.0001)	2.74 (2.01–3.73)	(<0.0001)

OR odds ratio CI confidence interval ^a^likelihood ratio test P_T_ *P* value for trend ^b^adjusted for all variables in table BMI body mass index eGFR estimated glomerular filtration rate ACEI angiotensin converting enzyme inhibitors ARBs angiotensin II receptor blockers Sep. September Oct. October

## Abbreviations

ACEI: Angiotensin converting enzyme inhibitor
AKI: Acute kidney injury
AKIN: Acute Kidney Injury Network
aOR: Adjusted odds ratio
ARB: Angiotensin II receptor blocker
BMI: Body mass index
CAP: Community-acquired pneumonia
CI: Confidence interval
CKD: Chronic kidney disease
CPRD: Clinical Practice Research Datalink
eGFR: Estimated glomerular filtration rate
HbA1C: Glycated-haemoglobin
HES: Hospital Episode Statistics
ICD: International Classification of Diseases
IMD: Index of Multiple Deprivation
KDIGO: Kidney Disease: Improving Global outcomes
OR: Odds ratio
